# Patients’ Comprehension of Mindfulness-Based Cognitive Behavioral Therapy in an Outpatient Clinic for Resistant Depression: A Cross-Sectional Study

**DOI:** 10.3389/fpsyg.2019.00271

**Published:** 2019-02-12

**Authors:** Michele F. Rodrigues, Carlos Campos, Luisa Pelucio, Izabel Barreto, Sergio Machado, Jose C. Appolinario, Antonio E. Nardi, Michelle Levitan

**Affiliations:** ^1^Treatment Resistant Depression Ambulatory, Psychiatry Institute, Federal University of Rio de Janeiro, Rio de Janeiro, Brazil; ^2^Center for Rehabilitation Research, School of Health, Polytechnic Institute of Porto, Porto, Portugal

**Keywords:** comprehension, mindfulness, cognitive therapy, depression, baseline

## Abstract

The current study investigated the comprehension of mindfulness-based cognitive behavioral therapy (MBCT) by patients with resistant depression at the Psychiatry Institute of the Federal University of Rio de Janeiro, Brazil. This was the first time the model was used in the institution to treat these patients. In this study, 45 patients were invited to participate in a baseline session of MBCT that consisted in the explanation of the model and experimental exercises conducted by two experienced therapists. Twenty eight patients accepted to participate. At the end of the intervention, the patients completed a self-administered questionnaire designed by our ambulatory to assess their understanding of the method’s goals. Patients with anxiety disorder was also accessed for group comparison. More than 75% of the patients rated the intervention as comprehensible and workable. Compared to patients with depression, patients with anxiety had a better understanding of the mindfulness framework (6.5%) and the meaning of cognitive behavioral therapy (17.6%). Patients that completed the intervention described the baseline session of MBCT as comprehensive and acceptable. These results may allow possible future developments in the practice of mindfulness as a treatment applicable in many condition and settings even in the Brazilian context.

## Introduction

Studies have shown that the collaborative alliance is a key factor in treatment outcome (Horvath and Symonds, 1991). The collaborative alliance changes the therapeutic process, improving patient-therapist dynamics (Bordin, 1979), including several key elements: confident collaboration, bonding, idealized relationship, dedicated patient, help received, and goals and tasks. This brief study addresses patients’ feelings about “goals and tasks” during a session of psychoeducation with MBCT for resistant depression (Hatcher and Barends, 1996; Segal et al., 2002).

The collaborative alliance is particularly important in mindfulness-based cognitive behavioral therapy (MBCT). MBCT is a special kind of CBT that emphasizes the importance of understanding the processes underlying the improvements in patients’ thoughts, feelings, and behaviors. The identification of negative thoughts and cognitive restructuring are taught since the beginning of the treatment (Jarrett et al., 2011).

Mindfulness-based cognitive behavioral therapy has been developed specifically to prevent relapse in patients with a history of recurrent major depressive episodes (Segal et al., 2002). According to the MBCT model, individuals with previous episodes of major depression differ from those without such a history in the patterns of negative thinking, in which small downward mood shifts are more likely to produce recurrence, because they activate patterns of self-deprecating depressogenic thinking similar to those that prevailed in preceding episodes (Williams et al., 2008). MBCT integrates aspects of CBT for depression into mindfulness-based stress reduction (MBSR). By teaching patients to become more aware of their thoughts, feelings, and bodily sensations, MBCT can mitigate depressive symptoms (Beck, 1979; Kabat-Zinn and Hanh, 2013). It is in indeed widely described in the literature how the dimensions of physical and mental health/disease elude patients awareness and are difficult to be integrated as co-occurrent (Grossman et al., 2004; Mannarini and Boffo, 2014). Hence, the MBCT can have a pivotal role in psychopathologies such as depression, in which the psychosomatic component is fundamental and co-occur with mental symptoms.

Various studies have assessed the factors associated with the impact of patients’ understanding of the rationale and techniques related to the type of psychotherapy they are enrolled in. In general, the presence of basic psychological processes such as learning, perception, and social interaction are identified as common factors related to successful psychotherapies (Ablon and Jones, 1999).

Until now, no study has investigated the factors of treatment adherence and change regarding the theoretical and practical approach of MBCT in the baseline session focusing on these data. In Brazil, the adoption of this model is still limited, so little is known about patients’ understanding and acceptance of the model, especially in resistant depression, where oftentimes patients have already tried different treatment modalities. Furthermore, because their cognitive impairment frequently impacts the expected evolution, it is difficult to assume that any treatment model will be fully understood.

Understanding the psychotherapy approach can impact the therapeutic adherence and change. The present study is aimed at investigating the impact of patients’ understanding of MBCT and its applicability to the Brazilian population. We thus conducted an educational session including practical exercises with patients seeking psychiatric treatment, who completed a self-administered questionnaire regarding their understanding of the model and belief in its efficacy.

By teaching patients to become more aware of their thoughts, feelings, and bodily sensations, MBCT can mitigate depressive symptoms (Beck, 1979; Kabat-Zinn and Hanh, 2013). It is in indeed widely described in the literature how the dimensions of physical and mental health/disease elude patients awareness and are difficult to be integrated as co-occurrent (Grossman et al., 2004; Mannarini and Boffo, 2014). Hence, the MBCT can have a pivotal role in psychopathologies such as depression, in which the psychosomatic component is fundamental and co-occur with mental symptoms.

## Methods

### Study Design

It was performed a cross-sectional analysis of the data related to the understanding of MBCT protocol by patients with depression and another group of anxiety’s patients. Both groups were compared and evaluated in parallel.

### Participants

A total of 45 patients referred to the Psychiatry Institute of the Federal University of Rio de Janeiro, Brazil, were invited to participate in the baseline MBCT session in the study after having their psychiatric consultation, and 28 agreed to participate. All patients were evaluated using the Mini International Neuropsychiatric Interview (MINI 5.0.0/DSM IV, 2000) applied by trained health professionals who were independent from the study for diagnostic assessment. Inclusion criteria for all patients were age between 18 and 60 years and diagnosed with depression and anxiety disorder. The sample excluded patients with schizophrenia or difficulties in reading and writing, evaluated when they were submitting their data in a form. All volunteers signed a free and informed consent form and followed instructions regarding the study’s procedures. The study was approved by the Institutional Ethics Committee of IPUB/UFRJ.

### Procedure

After the MINI interview, patients were invited to participate in a small group or alone in a baseline session of MBCT. Two psychologists with recognized specialization in cognitive-behavioral therapy, frequently users of meditation extensively studied the MBCT protocol (Teasdale et al., 2016) explained the model and conducted experimental exercises. All the stages were conducted in the same room. The first part consisted of the explanation of the cognitive, lasting 10 min. The points considered were: brief psychoeducation regarding the link between triggering situations, automatic thoughts, and dysfunctional attitudes (Beck and Beck, 2011).

Secondly, after confirming the patient’s understanding of the cognitive model, we explained the concepts of mindfulness and guided the patients through a short meditation exercise (“mindfully eating a raisin”), providing a mindful awareness experience by paying attention to the details and being present in the moment (body scan) (Segal et al., 2012), lasting 10 to 15 min. At the end of these interventions, patients completed a self-administered questionnaire on their understanding of the goals. A quiet room from the Depression Resistant Ambulatory was separated in advance to accommodate the patients in order not to be disturbed, in comfortable chairs and air conditioning.

### Instruments

We developed an instrument based on the specific rationale and strategies of the MBCT therapy (Teasdale et al., 2016), consisting of a 27-item self-report on acceptability, comprehension, and specific benefits of the model (Table 1). This table consisted of items adapted from a previous feasibility study (Campos et al., 2015; Kvillemo et al., 2016).

**Table 1:** Intervention acceptability questionnaire.

Intervention acceptability and comprehension		Yes	Maybe	No
*I was able to understand the mindfulness framework*	Depression	14 (82.4%)	3 (17.6%)	0 (0%)
	Anxiety	8 (88.9%)	0 (0%)	1 (11.1%)
*I found the raisin exercise helpful to the purpose of the section*	Depression	15 (88.2%)	2 (11.8%)	0 (0%)
	Anxiety	8 (88.9%)	1 (11.1%)	0 (0%)
*I felt comfortable to perform the mindfulness exercise*	Depression	15 (88.2%)	1 (5.9%)	1 (5.9%)
	Anxiety	8 (88.9%)	0 (0%)	1 (11.1%)
*I would be capable of performing mindfulness exercise at home*	Depression	13 (76.5%)	3 (17.6%)	1 (5.9%)
	Anxiety	8 (88.9%)	1 (11.1%)	0 (0%)
*I was able to understand the meaning of cognitive behavior therapy*	Depression	14 (82.4%)	3 (17.6%)	0 (0%)
	Anxiety	9 (100%)	0 (0%)	0 (0%)
*I would be capable of performing this type of writing exercise at home*	Depression	15 (88.2%)	2 (11.8%)	0 (0%)
	Anxiety	8 (88.9%)	1 (11.1%)	0 (0%)
*I found this type of treatment complicated*	Depression	2 (11.8%)	3 (17.6%)	12 (70.6%)
	Anxiety	0 (0%)	3 (33.3%)	6 (66.7%)
*I would recommend this type of treatment to someone with depression*	Depression	13 (76.5%)	4 (23.5%)	0 (0%)
	Anxiety	8 (88.9%)	1 (11.1%)	0 (0%)
*I would like to take part in this type of treatments*	Depression	11 (64.6%)	6 (45.3%)	0 (0%)
	Anxiety	5 (55.6%)	4 (44.4%)	0 (0%)
*I would be available to make this treatment once weekly*	Depression	7 (41.2%)	8 (47.1%)	2 (11.8%)
	Anxiety	*3 (33.3%)*	6 (66.7%)	0 (0%)
*Would you commit yourself to put time and effort into home practices and fill out questionnaires?*	Depression	9 (52.9%)	7 (41.2%)	1 (5.9%)
	Anxiety	*3 (33.3%)*	6 (66.7%)	0 (0%)
**Intervention specific benefits**				
*I found that treatment would be useful to me*	Depression	12 (70.6%)	5 (29.4%)	0 (0%)
	Anxiety	8 (88.9%)	1 (11.1%)	0 (0%)
*This treatment would help me handle with my negative thoughts*	Depression	14 (82.4%)	3 (17.6%)	0 (0%)
	Anxiety	8 (88.9%)	1 (11.1%)	0 (0%)
*Would this type of treatment reduce my anxiety?*	Depression	11 (64.7%)	6 (35.3%)	0 (0%)
	Anxiety	8 (88.9%)	1 (11.1%)	0 (0%)
	Anxiety	7 (77.8%)	1 (11.1%)	1 (11.1%)
*This type of treatment would improve my well-being*	Depression	10 (58.8%)	6 (35.3%)	1 (5.9%)
	Anxiety	8 (88.9%)	1 (11.1%)	0 (0%)


Mini International Neuropsychiatric Interview-Brazilian Version 5.0.0 – International Neuropsychiatric Interview: an instrument with a standard model of a brief structured interview (approximately 25 min) for the evaluation of the existence of Axis I psychiatric disorders according to the DSM-IV (Amorim, 2000).

All data were entered and analyzed using the software IBM SPSS Statistics for Windows, Version 20.0 (IBM Corp., Armonk, NY, United States).

## Results

### Sociodemographic Data

The sample included patients (*n* = 28) with a mean age of 40.7 years (*SD* = 14.66), 18 of whom were men; 53.5% had completed undergraduate education. Fifty percent of the patients had depressive disorder and 50% had anxiety disorder.

### Acceptability and Specific Benefits

According to the study, 60.7% of patients expressed interest in participating in this type of treatment and willingness to devote time and effort to home exercises and completion of questionnaires; 42.9% would be willing to undergo this treatment on a once-weekly basis.

Over 75% of the patients rated the intervention as understandable and feasible. Patients also stated that they would recommend this type of treatment to someone with depression (82.1%; Table 1).

### Intervention Characteristics

As for the intervention’s characteristics, 67.9% of patients felt more comfortable with meditation rather than writing their thoughts on paper (21.4%). Furthermore, 67.9% of patients reported owning a cell phone or other device to listen to the audio content, and 50% of patients preferred individual treatment, while 14.3% preferred group therapy.

### Depression and Anxiety Outcomes

We also divided patients according to primary diagnosis between depression and anxiety disorder. The two groups of patients had similar and positive answers in the acceptability and comprehension questionnaire (>70%). Meanwhile, the questions on commitment showed a lower response, where patients with depression expressed less commitment than patients with anxiety disorder (with a difference of some 20% in their commitment to invest time and effort in home exercises and completion of questionnaires).

Regarding the answers on specific benefits, there was wide variation between patients with depression and anxiety. Patients with anxiety disorder were more likely to consider the treatment useful, compared to patients with depression (18% difference), thus emphasizing that patients with anxiety disorder were more aware of their wellbeing than patients with depression (30%). Comparing overall and specific benefits, patients with depression were 25% more likely than patients with anxiety to consider the treatment beneficial overall. Meanwhile, patients with anxiety disorder were 24.2% more likely to acknowledge specific benefits in their symptoms (Table 1).

Concerning the intervention’s characteristics, patients with anxiety disorder had more devices like smartphones than patients with depression. Patients with anxiety disorder also had more difficulty with schedules than patients with depression (36.6% difference).

## Discussion

The aim of this brief study was to explore the impact of patients’ understanding of MBCT and its applicability to the Brazilian population. Patients who completed the intervention described it as comprehensible and acceptable. Prior studies on MBCT have found that most patients describe MBCT as acceptable, beneficial (Finucane and Mercer, 2006), and easy to understand (Phang et al., 2015).

One study of MBCT in a heterogeneous group of adult psychiatric outpatients applied the Credibility/Expectancy Questionnaire (Devilly and Borkovec, 2000) to 26 participants at the end of session two. This credibility questionnaire assesses patients’ willingness to recommend the treatment to others. The results indicated high confidence in recommending MBCT (Ree and Craigie, 2007); 82.1% of patients in the study reported that they would recommend the treatment to persons with depression.

In a previous study (Stafford et al., 2015), 95% of women completing the MBCT program reported high likelihood of continuing their mindfulness practice. We found that most patients approved the intervention and expressed their desire to commit to the treatment.

With regard to specific benefits, 75% of patients stated that “the treatment would be useful to me,” compared to a pilot study by Stafford in which 93% of patients found MBCT helpful (2014). Another interesting finding was that more than half of the patients reported discomfort in writing their thoughts on paper, which should be taken into consideration when developing the protocol. In fact, there is an informal consensus between cognitive therapists brought many times in meetings and congresses regarding a tendency of the Brazilian people to dislike and do less homework from therapy due to its cultural aspects. Brazilian population overall presents flexible rules, like tolerating delays and preferring spontaneity. Perhaps to some populations, the demand for homework should be revised.

Our results also identified differences between patients with anxiety versus depression, where patients with anxiety disorder were more likely to believe in specific benefits in their symptoms. These results are in accordance with the psychopathology of the depression, in which negative thoughts related to self-efficacy and a catastrophic perspective about the future are core features (Sugiura and Sugiura, 2016).

Although mindfulness-based treatment has always been typically the prerogative of the third wave of cognitivism, this can also be conjugated from a psychodynamic perspective (Bianco et al., 2016), thus allowing to argue some possible future developments in the practice of mindfulness as a treatment applicable in many condition and settings even in the Brazilian context.

A significant limitation to conclusions drawn from the study is the small sample size and a simple statistical analysis in the baseline MBCT session. Our group future research will focus on the results of psychotherapy in association with the results of this “baseline session,” enabling us to predict factors associated with success in this treatment modality. Furthermore, it is a goal of our group to amplify the use of meditation techniques to a wide broad population, and not to stay strict to diagnostic sample.

## Author Contributions

MR designed the study. MR, CC, LP, and IB collected, analyzed, and interpreted the data. AN, JA, and ML drafted and revisioned the manuscript. SM, AN, JA, and ML approved the final version of the manuscript.

## Conflict of Interest Statement

The authors declare that the research was conducted in the absence of any commercial or financial relationships that could be construed as a potential conflict of interest.
